# 1712. Reduction of Neonatal Methicillin-Susceptible *Staphylococcus aureus* Bloodstream Infections After Implementation of a *S. aureus* Screening and Decolonization Protocol

**DOI:** 10.1093/ofid/ofad500.1545

**Published:** 2023-11-27

**Authors:** Rachel Weber, Jeananne Wharry, Michael Malczynski, Jennifer Lehman, Leslie Caldarelli, Maureen K Bolon

**Affiliations:** Northwestern Memorial Hospital, Chicago, Illinois; Northwestern Medicine, Prentice Women's Hospital, chicago, Illinois; Northwestern Memorial Hospital, Northwestern University Feinberg School of Medicine, Chicago, Illinois; Northwestern Memorial Hospital, Chicago, Illinois; Northwestern University Feinberg School of Medicine; Ann & Robert H. Lurie Children's Hospital of Chicago, Chicago, Illinois; Northwestern University Feinberg School of Medicine, Chicago, Illinois

## Abstract

**Background:**

*Staphylococcus aureus (SA)* is an endemic, clinically important organism in Neonatal Intensive Care Units (NICUs). An 86-bed level III NICU at an academic medical center saw an increase in patients with methicillin-susceptible *SA* (MSSA) bloodstream infections (BSIs) from October-December 2020, as well as a cluster of patients with MSSA skin infections associated with intravenous catheters, which led to the intervention described.

**Methods:**

We created a multidisciplinary team and reviewed infection prevention measures in our NICU, national recommendations and published literature. Previous state, NICU patients were screened for methicillin-resistant *SA* (MRSA) on day of life 7 or upon admission from an outside hospital or home. On 11/1/2021, we implemented weekly screening cultures for MRSA and MSSA among all NICU patients, performed using a composite swab of the bilateral anterior nares, axilla, peri-umbilical area, and perianal area. Decolonization of *SA*-colonized patients was performed with mupirocin (MUP) and chlorhexidine gluconate (CHG) bathing. MUP was applied to nares twice per day for 5 days for all colonized patients and 5 days of CHG bathing was administered only to patients greater than 48 weeks postmenstrual age. Patients were decolonized up to 2 times. Incidence rates (IR) and incidence rate ratios (IRR) were calculated.

**Results:**

Between 11/1/2021 – 1/30/23, 1186 unique patients were screened with 177 unique patients colonized with MSSA and 9 unique patients colonized with MRSA. Of those colonized with MSSA, 126 (71.2%) patients were treated, with 123/126 treated with MUP only and 3/126 treated with MUP and CHG. Table 1 describes SA outcomes in the 15 month pre- and post-intervention periods. In the post-intervention period we observed a 73% decrease in MSSA BSIs (IRR, 0.27, 95% CI (0.05, 1.03)).

**Figure:**
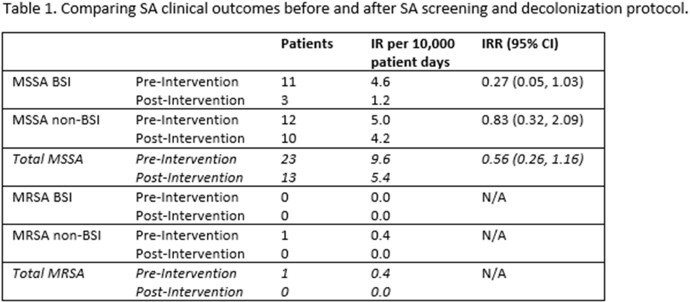

**Conclusion:**

Implementation of a *SA* screening and decolonization protocol in our NICU resulted in a statistically non-significant decrease in MSSA BSIs. Future work will seek to identify risk factors for colonization, factors associated with successful decolonization and genomic sequencing of MSSA isolates to characterize the spread of MSSA among neonates.

**Disclosures:**

**All Authors**: No reported disclosures

